# A Virtual Reality Game to Change Sun Protection Behavior and Prevent Cancer: User-Centered Design Approach

**DOI:** 10.2196/24652

**Published:** 2021-03-25

**Authors:** Caitlin Horsham, Ken Dutton-Regester, Jodie Antrobus, Andrew Goldston, Harley Price, Helen Ford, Elke Hacker

**Affiliations:** 1 School of Public Health and Social Work Queensland University of Technology Brisbane Australia; 2 QIMR Berghofer Medical Research Institute Brisbane Australia; 3 Excite Science Pty Ltd Brisbane Australia; 4 Preventive Health Branch Queensland Health Brisbane Australia; 5 Real Serious Games Pty Ltd Brisbane Australia

**Keywords:** virtual reality, gamification, primary prevention, health promotion, skin cancer, mobile phone

## Abstract

**Background:**

Public health sun safety campaigns introduced during the 1980s have successfully reduced skin cancer rates in Australia. Despite this success, high rates of sunburn continue to be reported by youth and young adults. As such, new strategies to reinforce sun protection approaches in this demographic are needed.

**Objective:**

This study aims to develop a virtual reality (VR) game containing preventive skin cancer messaging and to assess the safety and satisfaction of the design based on end user feedback.

**Methods:**

Using a two-phase design approach, we created a prototype VR game that immersed the player inside the human body while being confronted with growing cancer cells. The first design phase involved defining the problem, identifying stakeholders, choosing the technology platform, brainstorming, and designing esthetic elements. In the second design phase, we tested the prototype VR experience with stakeholders and end users in focus groups and interviews, with feedback incorporated into refining and improving the design.

**Results:**

The focus groups and interviews were conducted with 18 participants. Qualitative feedback indicated high levels of satisfaction, with all participants reporting the VR game as engaging. A total of 11% (2/8) of participants reported a side effect of feeling nauseous during the experience. The end user feedback identified game improvements, suggesting an extended multistage experience with visual transitions to other environments and interactions involving cancer causation. The implementation of the VR game identified challenges in sharing VR equipment and hygiene issues.

**Conclusions:**

This study presents key findings highlighting the design and implementation approaches for a VR health intervention primarily aimed at improving sun protection behaviors. This design approach can be applied to other health prevention programs in the future.

## Introduction

### Background

Australia has one of the highest rates of skin cancer in the world, with sunlight or ultraviolet radiation being the main risk factor for skin cancer [1]. Over the past three decades, Australia has successfully implemented world-class skin cancer prevention campaigns, such as Slip! Slop! Slap! and the SunSmart program using standard media channels, including posters, brochures, television, radio, and newspaper advertising [2,3]. The SunSmart program has led education, policy, and environmental intervention in schools, workplaces, and public settings, and these programs have raised public awareness and improved preventive behaviors among Australians, leading to a reduction in melanoma incidence in younger generations [4]. Despite this success, high rates of sunburn continue to be reported by young adults, with people aged between 18 and 34 years being 4 times more likely to report having a sunburn in the last 12 months than those aged over 65 years [5].

Although the reasons for youth sunburn rates are complex and multifactorial, one potential contributor is the communication platforms used to deliver sun safety messaging to this age group. Challenges include the limited reach of traditional media channels (television and print), which are not regularly consumed by modern youth, whereas digital platforms (social media) are highly competitive spaces for user attention. As such, developing innovative and appealing ways to reach people with health messages is a critical step in improving skin cancer prevention strategies.

An emerging approach that has been effective in influencing behavior change in other health-related campaigns is the combination of virtual reality (VR) and gamification. Gamification is “the use of game mechanics and experience design to digitally engage and motivate people to achieve their goals” [6] and thus may be used to encourage people to engage in healthy behavior. Examples of VR interventions to address health-related issues include pain distraction; treatment of psychiatric conditions, such as anxiety, phobias, eating disorders, and addiction; and assistance with physical rehabilitation [7-9]. VR has also been assessed for modifying health behaviors, such as smoking, diet, physical exercise, and compliance to prescribed treatment regimens [10-13]. Work in the cancer prevention setting has shown VR to enhance awareness of how to check for prostate cancer symptoms in men [14].

With regard to the user experience, a systematic review of randomized controlled trials using VR technology found that interventions were generally effective and well tolerated by users [13]. In addition, VR experiences have also been shown to improve recall compared with more traditional 2-dimensional video platforms watched on either desktop computers or tablets [15,16], and participants using VR were found to explore more of the environment than those provided with a desktop display [16].

However, although there is evidence to support the deployment of VR technology in health promotion programs, designing health interventions is challenging, and poor design choices can lead to interventions failing to meet their objectives [17]. One approach to overcome these hurdles is a user-centered design strategy. Here, content is developed using a multistage problem-solving process to assess how end users are likely to use a product and actively requires end user participation during the design process [18]. By involving end users and other stakeholders (such as individuals paying for the product, industry, and health authorities and government officials), uptake and use are increased and this ensures consistent messaging across all channels [19]. User-centered design can assist health interventions and programs that contribute to the development of more user-friendly, meaningful interventions for improving health outcomes.

### Objectives

This study aims to create a VR game that provides skin cancer preventive messaging. This study describes the design process, development pipeline, and implementation experiences.

## Methods

**Figure 1:**
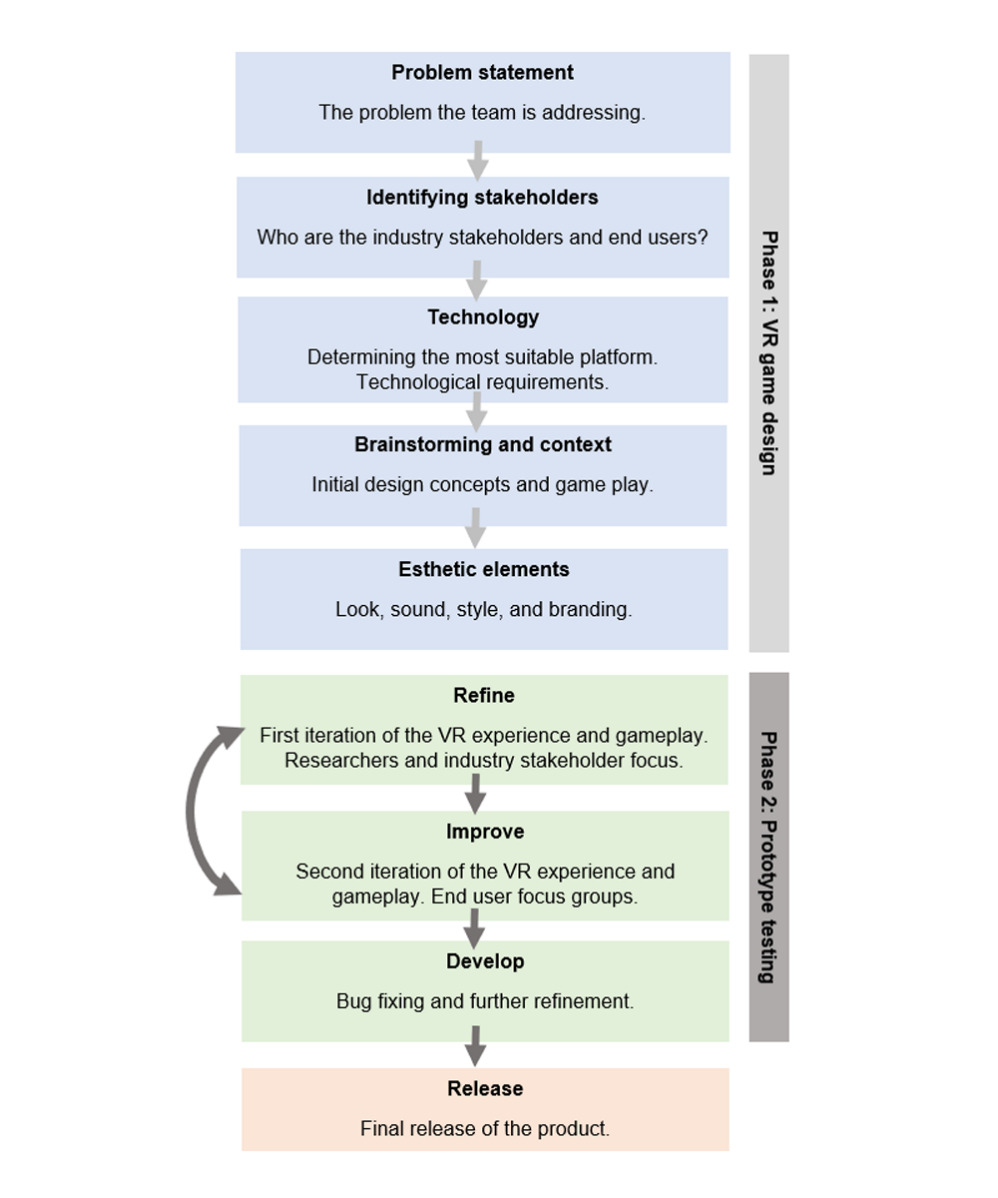
Flow chart of the design and development process. VR: virtual reality.

The overall process of developing the VR game included *phase 1: game design*, which involved defining the problem, identifying stakeholders, choosing the technology platform, brainstorming and context, initial development meetings, and designing esthetic elements, and *phase 2: prototype testing*, which involved pilot testing the VR experience with stakeholders and then end users in focus groups and interviews, with feedback incorporated into refining, improving, and further developing the VR experience (Figure 1).

### Phase 1: VR Game Design

#### Problem Statement

The team was addressing skin cancer prevention challenges in Australia. Despite previous campaigns, Australians continue to report high rates of sunburn, and people are increasingly turning away from traditional media consumption such as television and print; hence, novel approaches are required to reach this audience.

#### Development Team and Stakeholders

The development team comprised a multidisciplinary group including cancer researchers, software developers, science communicators, government health officers, and end users. Cancer researchers had expertise in cancer biology, clinical trial design, development and implementation, science communication, and testing of health technologies. The software developers had professional roles in the design studio *Real Serious Games*. The government health officers were experienced policy officers at the Preventative Health Branch. We specifically targeted end users aged 18 to 35 years who reported higher rates of sunburn; however, the game was designed to be suitable for all ages. These stakeholders contributed to the iterative design process and provided feedback on which aspects of the gamification development they liked and disliked.

#### Technology

During our first development team meetings, we discussed the challenges that arose when communicating cancer-themed information. A common difficulty encountered by stakeholders was the ability of target audiences to visualize and relate to the disease because of the often internal and microscopic scale of the disease. As such, the benefits of using VR technology as a communication platform were discussed.

One benefit of VR over other communication platforms is that VR allows users to be immersed in a simulated environment where they can interact with their surroundings using a headset and hand controllers. This was particularly desirable from the development teams’ perspective, allowing participants to be taken into the human body to visually see how cancer occurs at a macroscopic, first-person point of view. In addition, VR has been shown to be more effective for memory recall and behavior change than digital desktop delivery [16] and can be combined with gaming elements to provide an entertaining communication channel relevant to a youth audience.

For the reasons mentioned earlier and the fact that VR as a health promotion tool has been limited, we chose to use VR technology to engage users with health information. We specifically chose the Oculus Quest platform (Facebook Technologies LLC) to deliver the VR experience because of its versatility in field delivery (ie, a stand-alone unit that does not require connection to high-end computing hardware or room motion sensors to detect movement). Unity software, a cross-platform game engine, was used to develop the game (Unity Software Inc).

#### Brainstorming and Context and Esthetic Elements

During the brainstorming phase, there were discussions to incorporate multiple levels and scenarios with the VR game such as interactions between the skin and sunlight, cancer cells transporting through a blood vessel, and a metastatic lung site. However, health interventions often have budgetary restraints and confining the prototype game to one level, allowed for a focused approach while still remaining within the budget. This also enabled us to develop a minimum viable product to assess whether VR was a suitable platform for our target audience while remaining within the budget. Taking the abovementioned points into consideration, the development team decided that the best approach was to develop a first-person shooter game based inside the body, depicting cancer growth and the challenges encountered with late-stage therapy. The narrative journey was also built around minimizing production costs by reducing the need to create VR visual resources; instead, a black scene with voice-over could transition the user into the experience. However, esthetic elements were an important factor in the design to ensure that the user experienced a sensation of being transported inside the human body. Considerable resources were provided to ensure that the VR visual elements were anatomically correct and had an authentic look and style.

The VR experience we created begins with audio describing how a skin cancer has invaded your body (Multimedia Appendix 1), and the user is then transported into the lungs of a human body where a dark pigmented melanoma begins to grow. A shooter game play mechanism is used to fight the cancer using several treatments, including chemotherapy, targeted therapy, and immunotherapy (Multimedia Appendix 2). The hand controllers are used to target and destroy the cancer cells, whereas a proliferation algorithm determines how quickly the cells divide and replicate. As the game proceeds, the cancer cells become resistant, requiring the player to progressively escalate through the different treatments to *defeat* the growing cancer. This was purposefully designed to demonstrate the difficulties of treating late-stage cancer and to reinforce the importance of early detection and adoption of preventative health strategies. The game ends with a prevention message conveyed by the text *not everyone wins*, followed by *prevention is the best cure* and *protect your skin everyday*. The subtle health messaging accompanying the game is designed to provide an entertaining experience while learning more about skin cancer. In summary, the VR game focuses on the difficulties of treating metastatic skin cancer and illustrates to the user how simple prevention is and why it is important.

To embed behavior change–relevant cues, we adopted the Appeal, Belonging, Commitment (ABC) framework into our design process [20]. The integrated ABC framework combines elements of the health belief model, social cognitive theory, and transtheoretical model [21] and has previously been used successfully in the context of an interactive mobile phone game teaching antiviolence norms to young men [22]. The ABC framework can be used to assume that the user’s intention to perform sun protection will be influenced by the perceived value to the user (*Appeal).* It includes *Belonging* (socially based) about whether key people approve or disapprove of sun protection and motivation to behave in a way that gains their approval. Finally, it incorporates *Commitment* (stage of change), which needs to occur to ensure habitual behavior. This includes the belief that one has and can exercise a perceived likelihood of control over using sun protective behaviors. The VR game created a unique, compelling user experience designed to resonate and appeal to the user (Appeal). The game play encourages people’s intrinsic need for competition and is designed to build discussion among social groups about the experience and continue to shape social norms (Belonging). The messaging at the end of the game is designed to empower the participant to take responsibility for their sun protection behaviors and motivate improved sun protection habits in the future (Commitment).

### Phase 2: Prototype Testing

#### Refining With Stakeholders

In the first step, we tested the game play mechanisms and transition steps among a panel of industry stakeholders, including educators, science communicators, and government personnel (n=12). Each individual was asked to participate in a 1- to 3-minute VR experience, which included the chemotherapy treatment level. Focus groups were then undertaken on a range of topics, including enjoyment, esthetics, usability and game play design, safety, and future refinements. Notes were taken during the focus group feedback sessions by the moderator and findings reported back to the design team. No formal qualitative analyses were conducted.

Feedback from stakeholders emphasized safety aspects, and the VR game was designed as a sitting experience with visual elements designed in central fields of view to reduce head movement.

#### Improving With End Users

The focus groups and interviews were conducted with end users, all participants provided written informed consent, and the study was approved by the Queensland University of Technology Human Research Ethics Committee (Approval number: 1900001157). Participants were recruited via university emails and the social media platform Facebook during September (spring) in Australia.

The end users were asked to participate in one 60-minute focus group or interview, which included testing the 3- to 5-minute VR experience and providing feedback (Multimedia Appendix 3). The audio-recorded focus groups or interviews discussed a range of topics, including satisfaction with the experience, barriers to understanding the information, usability and game play design, esthetics, deployment opportunities, and safety during the experience. Thematic analysis was undertaken by one researcher (CH) following an iterative process of familiarizing oneself with data, generating initial codes, defining and naming themes, reviewing themes, and searching for themes.

Before the focus group, participants completed a web-based demographic survey and a follow-up survey 7 days after the VR experience. The post-VR survey asked if they experienced any of the following negative symptoms after use of the VR experience, including nausea or motion sickness; dizziness, disorientation, or impaired balance; altered or blurred vision or other visual abnormalities; eye strain or eye or muscle twitching; or headache. Participants were also given the opportunity to list any other symptoms experienced and were asked to provide additional details about their symptoms in an open-ended question.

Information on participants’ skin color, skin cancer history, and sun protection habits were collected. The sun protection habits index was used, which queries the frequency of 7 sun protective habits that are used when outdoors using a 4-point Likert scale (1=never or rarely, 2=sometimes, 3=usually, and 4=always) [23,24]. The overall sun protection index score is derived from questions including wearing a shirt with long sleeves, wearing a hat, wearing sunglasses, wearing sunscreen with a sun protection factor (SPF) of 15 or more on the face, wearing sunscreen with a SPF of 15 or more on other parts of the body, staying in the shade, and limiting time in the sun during midday hours.

## Results

### End User Focus Group Characteristics

In total, 18 participants completed the focus groups. Overall, 7 focus groups were conducted, ranging from 1-4 participants per group. Participants were mainly university educated (13/18, 72%), 67% (12/18) were female, and 61% (11/18) had never experienced VR before (Table 1). The participants’ ages ranged between 18 and 74 years, and more than half of the participants had sun-sensitive characteristics, such as fair skin that would burn moderately or severely after summer sun exposure (Table 1). Overall, 2 out of 18 (11%) participants had previously been diagnosed with skin cancer (both basal cell carcinoma). Participants completing the study used sun protection behaviors when outdoors sometimes to usually, with an average sun protection habits index score of 2.48 (SD 0.49; 1=never or rarely, 2=*sometimes*, 3=*usually*, and 4=always; Table 2. Female participants tended to report more frequent use of sunglasses and sunscreen on their face, whereas men reported the use of long sleeve shirts and limiting time outdoors during midday hours (Table 2). We observed a trend for increased sun protection behaviors among participants aged ≥36 years compared with younger participants aged 18 to 35 years, although this was not significant (two-tailed *t* test *P*=.08; Table 2).

**Table 1:** End user focus group characteristics.

Characteristic			Baseline value (N=18)
Age (years), range			18-74
**Gender, n (%)**			
	Female	12 (67)	
	Male	6 (33)	
**Skin color, n (%)**			
	Very fair or fair	10 (56)	
	Medium	6 (33)	
	Olive or brown	2 (11)	
**Education, n (%)**			
	High school or leavers certificate	3 (17)	
	Trade, technical certificate, or diploma	2 (11)	
	University degree	13 (72)	
**Skin burn in strong summer sun for 30 minutes without protection, n (%)**			
	My skin would not burn at all	1 (6)	
	My skin would burn lightly	6 (33)	
	My skin would burn moderately	9 (50)	
	My skin would burn severely	2 (11)	
**Skin tan in strong sun without protection, n (%)**			
	My skin would not tan	2 (11)	
	My skin would tan lightly	2 (11)	
	My skin would tan moderately	11 (61)	
	My skin would tan deeply	3 (17)	
**Previously diagnosed with skin cancer, n (%)**			
	Yes	2 (11)	
	No	15 (83)	
	Unsure	1 (6)	
**Previously experienced virtual reality, n (%)**			
	Yes	6 (33)	
	No	11 (61)	
	Unsure	1 (6)	

**Table 2:** End user focus group sun protection habits (N=18).

Sun protection habit	All participants, mean (SD)	Female (n=12), mean (SD)	Male (n=6), mean (SD)	Age <35 years (n=9), mean (SD)	Age >36 years (n=9), mean (SD)
Overall SPH index^a^	2.48 (0.49)	2.55 (0.46)	2.36 (0.56)	2.29 (0.44)	2.68 (0.47)
Wear a shirt with long sleeves	2.39 (0.78)	2.33 (0.78)	2.50 (0.84)	2.33 (0.87)	2.44 (0.73)
Wear sunglasses	2.89 (1.02)	3.08 (0.90)	2.50 (1.23)	2.78 (1.09)	3.00 (1.00)
Stay in the shade	2.72 (0.46)	2.75 (0.45)	2.67 (0.52)	2.67 (0.50)	2.78 (0.44)
Limit your time in the sun during midday hours	2.83 (0.92)	2.75 (0.87)	3.00 (1.10)	2.56 (0.88)	3.11 (0.93)
Wear a hat	2.00 (1.09)	2.08 (1.08)	1.83 (1.17)	1.78 (0.97)	2.22 (1.20)
Wear sunscreen with an SPF^b^ of 15 or more on your face	2.50 (0.99)	2.67 (0.89)	2.17 (1.17)	2.11 (0.78)	2.89 (1.05)
Wear sunscreen with an SPF^b^ of 15 or more on other parts of your body	2.06 (0.94)	2.17 (0.84)	1.83 (1.17)	1.78 (0.83)	2.33 (1.00)

^a^SPH Index: The overall sun protection habits index score is derived from items including wear a shirt with long sleeves, wear sunglasses, stay in the shade, limit your time in the sun during midday hours, wear a hat, wear sunscreen with an SPF of 15 or more on your face, wear sunscreen with an SPF of 15 or more on other parts of your body.

^b^SPF: sun protection factor.

### End User Focus Group Side Effects

During the VR experience, the participants remained seated and were not required to walk around. This prevented participants from losing balance or slipping, thereby reducing trip hazards. Overall, 2 out of 18 (11%) participants reported that they felt nausea during the VR experience; this was extended for 1 of those participants, who reported nausea and headache 24 hours after the VR experience. Nausea may become more apparent if the headset is fitted incorrectly or has a poor focus. It is important to fit the headset correctly, even for short experiences. Some participants reported that the headset felt heavy, was slightly out of focus, or was too loose. In addition, 1 participant identified that they had a sore neck if they looked up often and that they would prefer not to have too much content in their peripheral vision.

### End User Focus Group Qualitative Analysis

Qualitative analysis of participants’ discussions resulted in the identification of several themes, which are described below and detailed in Table 3.

**Table 3:** End user focus group qualitative analysis themes and descriptions.

Theme	Description	Participant quotes
Engaging and appealing to play	The VR^a^ experience was described as “fun,” “enjoyable,” “hands-on,” “enlightening,” “insightful,” “impactful,” “immersive,” “rewarding,” “informative,” “cool,” “illustrative,” “informative,” “serious,” “awesome,” “powerful,” “unique,” and “awe inspiring.” Participants prefer VR over other presentation formats, such as videos or brochures. Participants identified being bored or distracted watching videos, and there was a better connection to the environment in the 3-dimensional immersion.	“To actually look at the cancer at a cellular level...it was awesome.” (Male, 36 years) “Different way I guess to learn about cancer, but to see cancer, how it grows, and how therapy can be ineffective.” (Female, 30 years) “The skin cancer message got across so much better when you felt like you were in it [the human body].” (Female, 26 years) “It’s [VR] much more powerful than a brochure.” (Female, 64 years)
Competition and challenge to beat cancer	Participants observed how the cancer could grow and change.	“...makes you think how quickly they [cancer] grow.” (Female, 27 years) “It’s a good indication of how quickly something like that [cancer] can progress.” (Female, 30 years) “I think it was fun, but I think you also did see the point in there about the cells dividing and how much of a battle it [cancer] really is.” (Male, 47 years)
Simplifying complex information	The information is displayed in a simple and easily understandable way.	“I loved how it actually broke down what was a very complex problem, into a very simple idea.” (Male, 36 years)
Promotes discussion	Participants would talk to others about the VR experience. It was identified as a good education tool for children.	“It would be something that kids would run home and talk to their parents about.” (Female, 44 years) “Can someone take a photo I want to put it on Twitter.” (Female, 30 years)
Stronger connection to cancer prevention	Participants would like more context-relevant visual cues on prevention to show the journey of a cancer cell from where it originates. Suggestions include starting the game outside the body with an individual being sunburnt, showing the response of skin cells to sunlight and how this leads to skin cancer.	“You do lose a little bit of the skin cancer – sun protection message because you are so excited about immunotherapy and the different cancer therapies.” (Female, 46 years) “I don’t draw the link then to put a hat on... so there needs to that better link.” (Female, 40 years) “The game would be better if it was more of a journey. ...You didn’t really know it was about skin cancer until the end.” (Male, 47 years)

^a^VR: virtual reality.

#### Engaging and Appealing to Play

Participants found the game was engaging, simplified complex information, and provided a unique gamification experience. All participants (n=18) reported that they liked the VR experience and that it was appealing and engaging. Of the 18 participants, 16 said that they would play the game again. Participants were asked whether they would prefer the VR experience over the same content provided in a video format. Most participants preferred the VR format because they felt like they were in the body, and it was more hands-on and engaging.

#### Competition and Challenge to Beat Cancer

The proliferation of cancer cells was created using an algorithm that can be increased or decreased to change the speed of growth. Most participants were not too overwhelmed when the cancer was growing during the different treatments. The cancer cells were designed to continually grow out of control, and some participants identified that it felt stressful and unachievable to fight against. One participant commented that this is an accurate representation of how it may feel to fight against cancer in real life. Most participants felt like they were winning, especially toward the end of the game during the immunotherapy stage.

A participant suggested that a scoreboard may help show how well they were progressing or playing. However, there was no consensus regarding having a scoreboard, as some participants liked the idea, whereas others said it may take away from the seriousness of the issue at hand and would not add value. One participant said that it would be difficult to have a metric or score that would be a suitable indication of a real-life scenario. The experience was seen primarily as educational, rather than just a game to win. If a scoreboard was to be included, participants discussed preferences for incorporating a bar showing the effectiveness of treatments, which would use a word scale such as high or low rather than a numbered scoreboard. Including a message on the scoreboard at the end of the game showing the cancer is in remission was also suggested and supported by participants.

#### Simplifying Complex Information

Most participants identified that the experience depicted complex information in a simple and easy-to-understand way.

#### Promote Discussion

All participants said they would share the VR experience with their friends or family.

#### Stronger Connection to Cancer Prevention

Participants identified that the link between skin cancer prevention and fighting the cancer cells was not immediately clear until the end of the experience when this was depicted via audio and text on the screen stating *prevention is the best cure* and *protect your skin everyday*. A few participants suggested that the main message of the VR experience was learning about the different types of cancer treatment, rather than what causes cancer and how to prevent cancer.

Most participants would like to have a debrief after the VR experience to learn more information, and some participants identified that the focus group discussion enhanced their learning from the game. During the focus group, participants were provided with a debriefing A3 poster, which explained the different treatments used in the game (Multimedia Appendix 4). If the VR experience was deployed during an event in a public setting, providing debriefing information using a brochure, poster, or quick response code linking to a website was suggested. If users were to download the game and play at home on their headsets, it was suggested that the debriefing material could be delivered via email or a website.

Participants suggested that the prevention message could be stronger and depicted visually (rather than text alone), for example, showing an individual in the sun, zooming in to a mole on the human body, and then showing how a mole can transition to cancer and spread to other organs. Participants supported the concept of a multistage game transiting through levels, which highlight the journey of a cancer cell. The current VR experience was 3 to 5 minutes, and most participants would prefer a longer VR experience (on average approximately 10 minutes). Some participants qualified they would only play longer if the experience involved multiple levels or stages and continued to be interactive (ie, not just listening to content). All participants were interested in further content depicting the interactions between sunlight and the skin. Most participants were interested in viewing the journey of the cancer cell, as it was transported through the body, and some participants were interested in other treatment types such as radiotherapy. Extending the game into a multistage game could be part of a future project; however, balancing the health impact and return on investment needs to be evaluated with a cost-effectiveness analysis approach to determine the overall health benefit of a longer extended VR intervention. Future research will aim to evaluate whether this VR game can change perceptions and behavior around sun safety in people.

#### Esthetics and User Functionality

The esthetic elements incorporated into the VR experience were further discussed with the participants. All participants reported that they clearly identified the cancer character as a villain. Most participants found the voice satisfactory or would like the cancer voice to be darker or scarier (Multimedia Appendix 1). One participant commented, “I think it got the scary message across.” However, one participant thought the voice was too dark and said they would prefer not to have the emotion within the voice, and another participant identified it was slightly difficult to understand the darker voice.

Helpful information was provided throughout the experience, including a progress bar and pop-up game play information. Some participants understood the progress bar; however, those not familiar with gaming were unaware of its function. The treatment shooter was placed at the bottom of the game play screen and had an accompanying pop-up instruction (Multimedia Appendix 2). However, once the trigger was pressed, the treatment instructions disappeared, and some participants reported not seeing the instructions, as they eagerly pressed the trigger to commence game play. A treatment assistant character provided audio instructions and a narrative guide throughout the experience. Only 1 participant reported feeling lost at the start and would have liked more of an introduction commenting “It was clear that they needed to shoot the targets, but I did not understand why*.*”

## Discussion

### Addressing Challenges

Implementation challenges encountered by the research team while testing the VR headsets included maintaining power to equipment and hygiene issues when sharing VR headsets.

#### Charging Time and Batteries

If the VR headsets were used for more than 3-4 hours, recharging was required. If deployed at events in public settings, access to electrical supply or portal-powered charging stations must be considered to charge the VR equipment. The charging time for VR headsets was also more than 1 hour, which meant that additional VR headsets were required so that equipment could be rotated between charging and use. Hand controllers also require AA batteries, which may require replacement.

#### Hygiene and Headset Sharing

During the stakeholder testing (before the COVID-19 pandemic), we noticed that individuals who wore makeup on their face were transferring cosmetics onto the VR headsets, making them difficult to clean. We implemented disposable VR masks for every use, not just if the participant preferred to use one. Each mask is single use, and it is fitted over the ears and across the eyes before a headset is placed to prevent the participant’s facial skin from touching the VR headset.

The end user focus groups were conducted during the COVID-19 pandemic, and very low infectious rates were observed during the testing period. Extra precautions were implemented to maintain strict hygiene standards and prevent the spread of infectious diseases. Additional protocols included the headsets being wiped down using antibacterial wipes after each use as well as hand sanitizers being used by the participants both before and after using the VR equipment. Participants were reminded via email the day before the interview or focus group not to attend if they were feeling unwell. Staff members wore gloves when sanitizing the headsets, and social distancing was maintained during the end user focus groups, with chairs placed 1.5 m apart (Multimedia Appendix 3). This VR experience did not require much space, as it was a sitting experience; however, social distancing would have been much more challenging if participants were required to stand and move around a room.

We also noticed that when using the headset in a hot environment such as in Australia during spring, participants may sweat while using the equipment, which then transfers onto the VR equipment, making it undesirable for sharing. This also reinforced the need for cleaning protocols when sharing VR equipment.

#### Deployment Challenges

Although optimism for VR remains high, consumer adoption remains low, with only 11% of adults in the United States owning a VR headset [25]. Cost, a lack of games, and worries about motion sickness are reported as barriers to uptake [25]. In our study, 22% (4/18) of participants currently owned a VR headset, and only 3 of the 14 participants who did not own one reported that they might purchase one in the future. VR headsets are not widely available in the community, and participants reported that the VR experience was novel and exciting. Research has found high levels of public interest toward VR in the health care setting [26], and meta-analysis has shown that VR training interventions improve health professional education knowledge and skills [27]. Participants discussed settings where a VR headset would be particularly useful and identified hospitals and clinics as well as educational settings such as schools. A few participants mentioned that the VR experience was a good description of what is happening inside the body of a cancer patient and might be helpful for cancer education in hospitals. A previous study has illustrated that the use of relaxing VR content shown to patients with skin cancer before undergoing complex surgery reduced anxiety [28]. The ability to deploy VR experiences within health care settings is limited by access to VR equipment, and the opportunity for patients to access engaging VR health content is still futuristic.

With personal ownership of VR equipment low compared with smartphone devices, the audience reach for VR content is reduced compared with social media or app platforms. However, VR content can be reached by international markets through web-based stores, providing scalability for VR health interventions. For example, a free VR relaxation game had more than 40,000 unique users during a 2-year period, illustrating the potential scale and reach [29].

It should also be noted that VR content can often be ported into a 2-dimensional desktop PC experience. Although this undoubtedly increases the accessibility at this point in time, this approach may reduce the impact, engagement, and recall of the target audience [15,16]. These effects may also be amplified in our target audience of 18- to 35-year-olds, whose attention is highly competitive in the digital landscape. Assessing the longevity and impact of delivering sun protection messaging in a 2-dimensional versus 3-dimensional environment remains out of scope for this study and should be assessed in the future. In any case, a health intervention that uses a VR game to engage young adults will need a strong promotion strategy to successfully reach large audiences.

Finally, the participants in this study were a convenience sample, mostly female, and highly educated, which may limit the generalizability of the study findings.

### Conclusions

Health messaging requires novel and engaging strategies to be effective in changing behavior and preventing cancer. We undertook a two-phase design approach to create a VR game, which immersed the player inside the body while the cancer grew. This design model allowed for refinement and improvements to be included throughout the process. The end user feedback identified improvements, which included extending the experience to a longer multistage game with visual transitions to other environments and interactions involving cancer causation. The implementation of the VR game identified challenges in sharing VR equipment and hygiene issues. Barriers to the deployment of VR health interventions at a population level were also recognized. This case study used an immersive VR technology platform and illustrated the design approach to create a cancer prevention message.

## Appendix

Multimedia Appendix 1 Virtual reality narrative. This audio file highlights the cancer voice and tone, and it also provides the starting narrative that was used to position the player inside the body where the interactive game began.

Multimedia Appendix 2 Virtual reality chemotherapy treatment. The top panel depicts the chemotherapy shooter, which was placed at the bottom of the screen and had accompanying instructions on use. The bottom panel shows how chemotherapy destroys the cancer cells.

Multimedia Appendix 3 A focus group session. The sessions with end users were conducted in a large soundproof room to ensure that several people could concurrently complete the experience, which allowed participants to commence the focus group discussion together as a group after completing the virtual reality experience.

Multimedia Appendix 4 Debriefing information poster providing an explanation of the gameplay experience and linking health facts.

## Abbreviations

ABC: Appeal, Belonging, Commitment
SPF: sun protection factor
VR: virtual reality
