# Supplementary figures and images for: A Virtual Reality Game to Change Sun Protection Behavior and Prevent Cancer: User-Centered Design Approach
Source: JMIR Serious Games. 2021 Mar 25;9(1):e24652. doi: 10.2196/24652 (PMC8294638; doi:10.2196/24652)

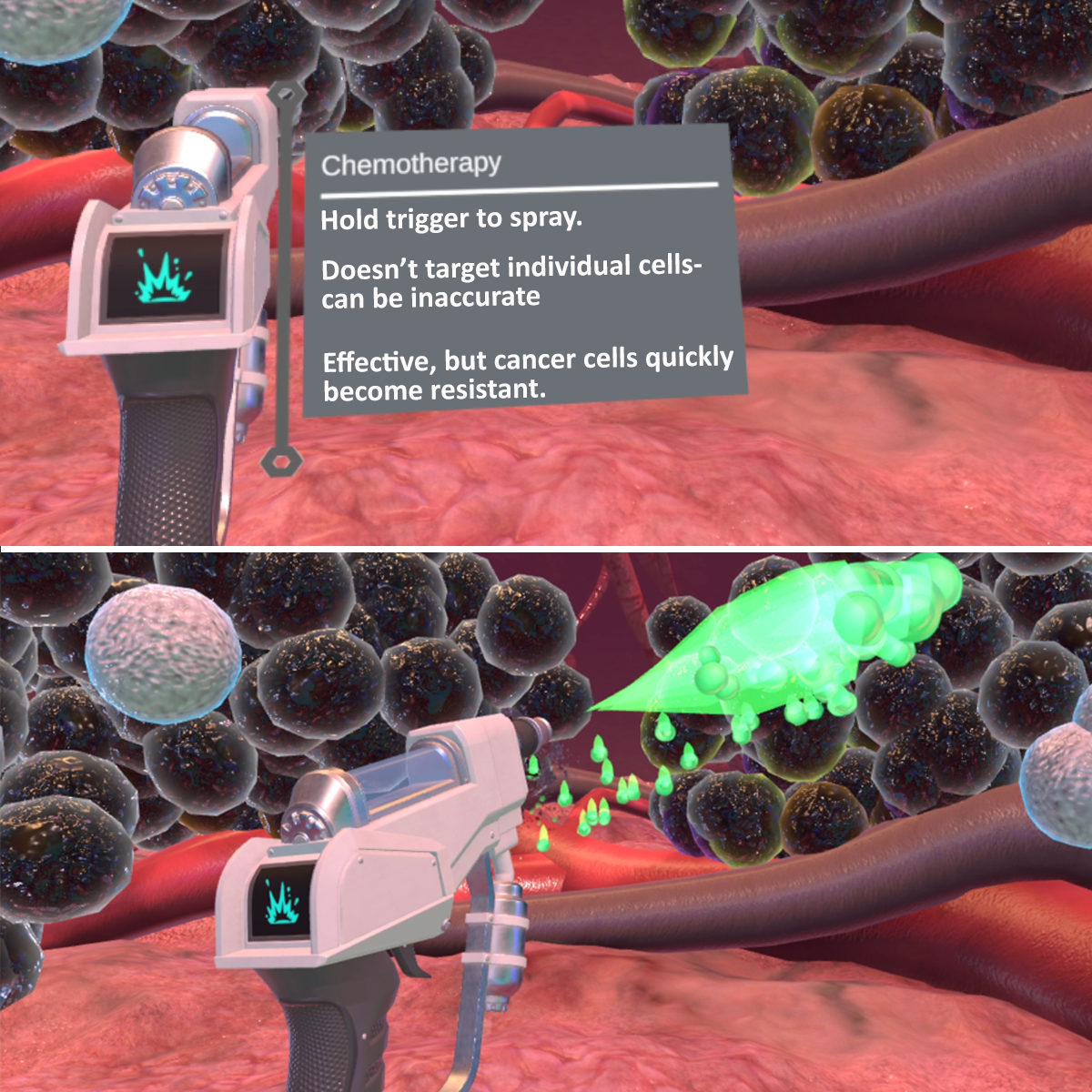

Supplement: Multimedia Appendix 2 [file games_v9i1e24652_app2.png]

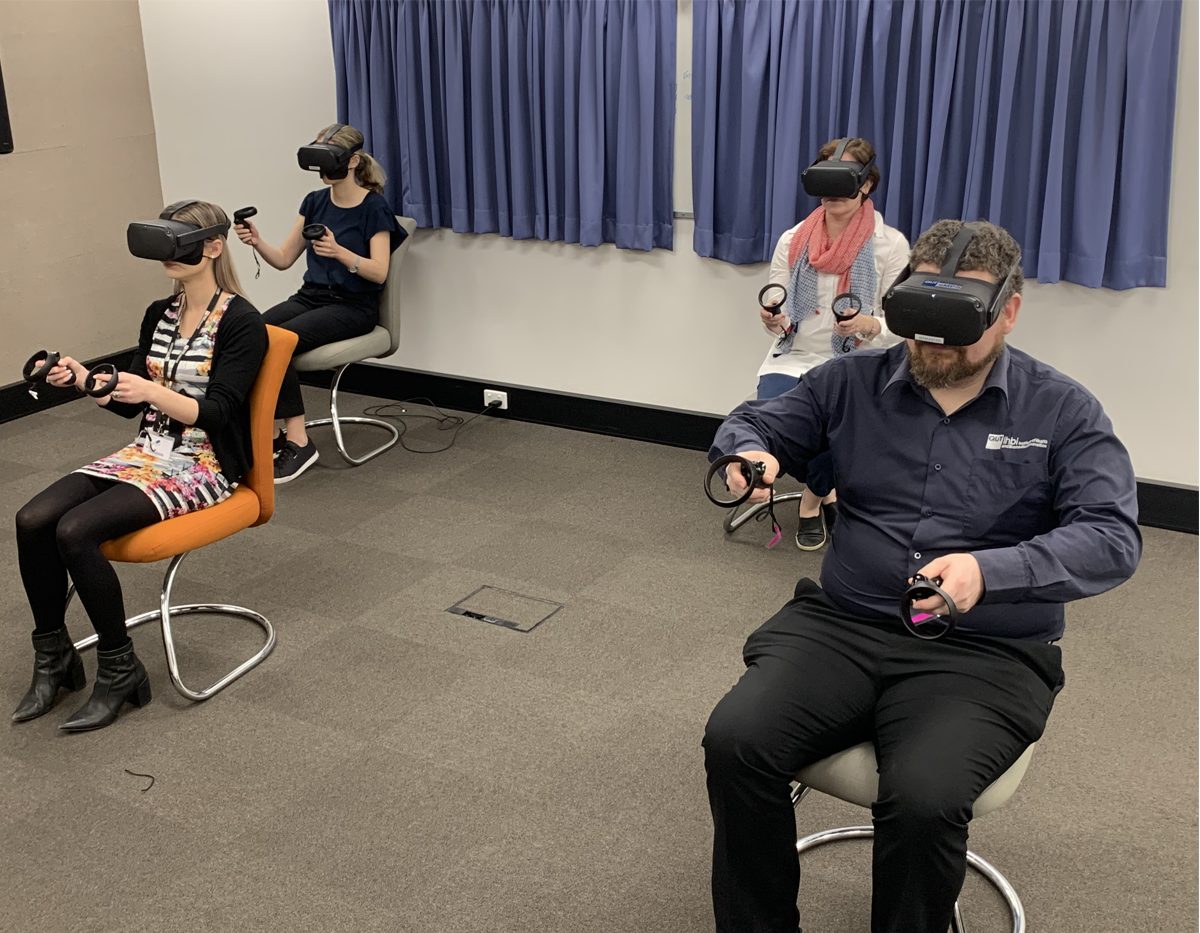

Supplement: Multimedia Appendix 3 [file games_v9i1e24652_app3.png]
