# Supplementary material for: A Virtual Reality Game to Change Sun Protection Behavior and Prevent Cancer: User-Centered Design Approach
Source: JMIR Serious Games. 2021 Mar 25;9(1):e24652. doi: 10.2196/24652 (PMC8294638; doi:10.2196/24652)
Supplement: Multimedia Appendix 4 [file games_v9i1e24652_app4.pdf]

A NEW WAY TO SEE CANCER !

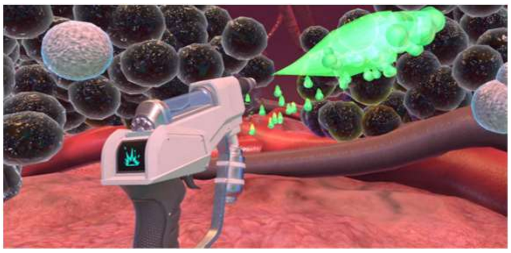

EXPLORE VIRTUAL TREATMENTS

SKIN CANCER

Every sunburn increases your risk of developing skin cancer.

Cancer cells can invade your blood system and appear in other areas such as the lungs. Once there, the cancer starts growing and a combinations of treatments must be used to fight the cancer cells.

CHEMOTHERAPY

Can destroy the cancer cells as they grow and duplicate. This treatment is effective at destroying cancers, but can also damage your body. Cancers can become resistant and continue to further invade the body.

TARGETED THERAPY

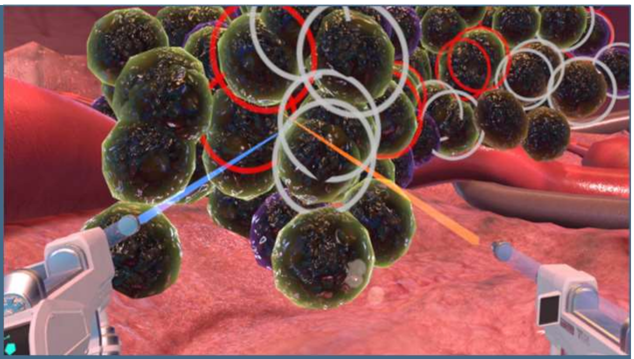

Can target and destroy genetically matching cancer cells. This treatment only targets cancer cells carrying a specific mutation, which are shown as yellow cancer cells. Targeted therapy only works against one mutation and resistant cancer cells can continue to grow.

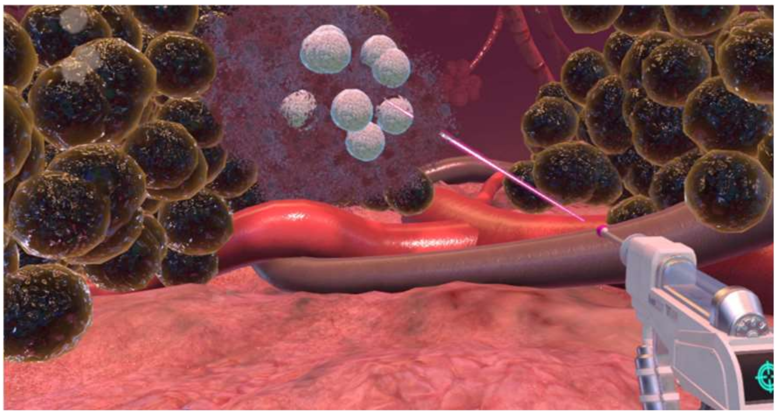

IMMUNOTHERAPY

Exposes cancer cells to your immune system to destroy them. Normally cancer cells hide from the immune system and this treatment decloaks cancer cells so nearby immune cells can attack and kill the cancer.

This project has been a partnership between QUT, Queensland Health, Real Serious Games and Excite Science Communications. This project was supported by Advance Queensland Fellowship funds.

QUT
